# Anaerobic oxidation of methane: an “active” microbial process

**DOI:** 10.1002/mbo3.232

**Published:** 2014-12-22

**Authors:** Mengmeng Cui, Anzhou Ma, Hongyan Qi, Xuliang Zhuang, Guoqiang Zhuang

**Affiliations:** Key Laboratory of Environmental Biotechnology, Research Center for Eco-Environmental Sciences, Chinese Academy of SciencesBeijing, 100085, China

**Keywords:** Anaerobic methanotrophic archaea, anaerobic oxidation of methane, *Candidatus* Methanoperedens nitroreducens, *Candidatus* Methylomirabilis oxyfera, methane

## Abstract

The anaerobic oxidation of methane (AOM) is an important sink of methane that plays a significant role in global warming. AOM was first found to be coupled with sulfate reduction and mediated by anaerobic methanotrophic archaea (ANME) and sulfate-reducing bacteria (SRB). ANME, often forming consortia with SRB, are phylogenetically related to methanogenic archaea. ANME-1 is even able to produce methane. Subsequently, it has been found that AOM can also be coupled with denitrification. The known microbes responsible for this process are *Candidatus* Methylomirabilis oxyfera (*M. oxyfera*) and *Candidatus* Methanoperedens nitroreducens (*M. nitroreducens*). *Candidatus* Methylomirabilis oxyfera belongs to the NC10 bacteria, can catalyze nitrite reduction through an “intra-aerobic” pathway, and may catalyze AOM through an aerobic methane oxidation pathway. However, *M. nitroreducens*, which is affiliated with ANME-2d archaea, may be able to catalyze AOM through the reverse methanogenesis pathway. Moreover, manganese (Mn^4+^) and iron (Fe^3+^) can also be used as electron acceptors of AOM. This review summarizes the mechanisms and associated microbes of AOM. It also discusses recent progress in some unclear key issues about AOM, including ANME-1 in hypersaline environments, the effect of oxygen on *M. oxyfera*, and the relationship of *M. nitroreducens* with ANME.

## Introduction

Methane is the second most abundant greenhouse gas after carbon dioxide (CO_2_), which accounts for 14% of global greenhouse gas emissions (EPA 2006). The concentration of methane in the atmosphere has increased ∼2.5 times than the preindustrial level, rising from 720 ppb in 1750 to 1803 ppb in 2011 (Hartmann et al. 2013). Although the methane concentration in the atmosphere is lower than the CO_2_ concentration (391 ppm), methane is 25-fold more effective in trapping heat in the atmosphere than CO_2_ on a per-molecule basis (IPCC 2007). Methane contributes to ∼30% of the anthropogenic warming, with the radiative forcing of 0.48 Wm^−2^ in 2011 (Myhre et al. 2013). After maintaining a relatively stable level for approximately a decade in the 1990s, the atmospheric methane concentration began to grow in 2007 (Hartmann et al. 2013). The concentration of methane in the atmosphere is determined by the balance of sources and sinks. The anaerobic oxidation of methane (AOM) is an important sink of the atmospheric methane concentration (Conrad 2009), which significantly impacts global warming. In marine sediments, the total amount of gas hydrates is up to 150–3000 times the atmospheric methane concentration (500,000–10,000,000 Tg CH_4_) (Reeburgh 2007). Fortunately, most of the mobilized CH_4_ is consumed through anaerobic methane oxidation, with a consumption rate of approximately 70–300 Tg CH_4_ year^−1^. Without this process, there would be an additional 10–60% of CH_4_ in the atmosphere (Conrad 2009).

**Figure 1:**
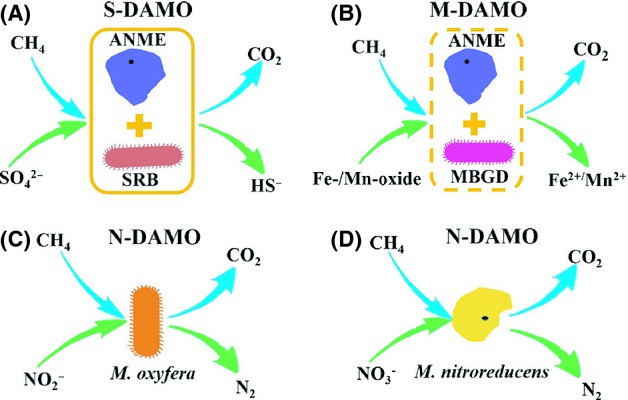
Three different models of anaerobic methane oxidation (AOM) depending on the different electron acceptors: (A) sulfate-dependent anaerobic methane oxidation (S-DAMO); (B) metal ion (Mn^4+^ and Fe^3+^)-dependent anaerobic methane oxidation (M-DAMO); and (C, D) nitrate/nitrite-dependent anaerobic methane oxidation (N-DAMO). ANME, an anaerobic methanotrophic archaea; SRB, sulfate-reducing bacteria; *M. oxyfera*, *Candidatus* Methylomirabilis oxyfera; *M. nitroreducens*, *Candidatus* Methanoperedens nitroreducens; MBGD, marine benthic group D.

AOM was first discovered in 1976 and is coupled with sulfate reduction in marine sediments (Reeburgh 1976, 1980). However, the responsible microorganisms for this process were actually identified ∼20 years later (Hinrichs et al. 1999; Boetius et al. 2000; Bian et al. 2001). In 2006, a new AOM process named nitrite-dependent anaerobic methane oxidation (N-DAMO) was reported; this process is coupled with denitrification (Raghoebarsing et al. 2006). It was shown that nitrate could also be an electron acceptor of AOM in addition to nitrite (Haroon et al. 2013). Likewise, Beal et al. (2009) suggested that AOM is coupled with the reduction of manganese (Mn^4+^) and iron (Fe^3+^) in marine sediments. Overall, there are three different processes of AOM depending on the different electron acceptors: sulfate-dependent anaerobic methane oxidation (S-DAMO) (Fig.1A), nitrate/nitrite-dependent anaerobic methane oxidation (N-DAMO) (Fig.1C and D), and metal ion (Mn^4+^ and Fe^3+^)-dependent anaerobic methane oxidation (M-DAMO) (Fig.1B). This review summarizes the biochemistry and microbiology of these three AOM processes, including the mechanisms and distribution of AOM processes, the responsible microbes, and their peculiar properties. Moreover, this review also discusses several key issues about the recent progress of AOM that are still unclear.

## Sulfate-Dependent Anaerobic Methane Oxidation

### Mechanism and distribution of S-DAMO

S-DAMO (eqs. 1–3) was discovered during geochemical studies conducted ∼40 years ago; these studies first suggested the process of AOM (Reeburgh 1976). In the following decades, more evidences are accumulated indicating that S-DAMO is mainly distributed in marine environments (Reeburgh 1976, 2007; Gal'chenko et al. 2004; Durisch-Kaiser et al. 2005; Orphana et al. 2005; Treude et al. 2007) and in freshwater environments (Murase and Kimura 1994; Grossman et al. 2002; Eller et al. 2005; Alain et al. 2006; Smemo and Yavitt 2007; Miyashita et al. 2009). These studies demonstrated that S-DAMO exists widely in natural ecosystems where it may play an important role in the biogeochemical cycling of carbon and sulfur.

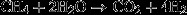
1

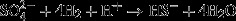
2

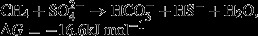
3

However, the exact metabolic mechanism of S-DAMO still remains unclear. Hoehler et al. (1994) proposed that methane is oxidized via a reversed methanogenesis (a reversal of CO_2_ reduction) under anoxic conditions. The product of methane oxidation is hydrogen (H_2_), which is used by sulfate-reducing bacteria (SRB) to yield bicarbonate and sulfide, with methane and sulfate in the ratio of 1:1 (Nauhaus et al. 2002, 2005). Many proteomic and genomic studies have been documented which support for the reverse methanogenesis hypothesis. A new nickel protein (Ni-protein I) was extracted from microbial mats suited for biochemical studies of AOM; this protein may play a catalytic role in AOM and is similar to the nickel cofactor F_430_ of methyl coenzyme M reductase (MCR), the terminal enzyme of methanogenesis (Krüger et al. 2003; Mayr et al. 2008). Scheller et al. (2010) discovered a purified MCR from a methanogen that could cleave the particularly strong C-H bond of methane to form methyl coenzyme M. The metagenome and mRNA expression analyses of ANME-1 (anaerobic methanotrophic archaea) support the reverse methanogenesis hypothesis (Meyerdierks et al. 2010). Four groups of the *mcrA* gene (coding for the α-subunit of MCR) were shown to correspond to the ANME community (Hallam et al. 2003). Based on observations of the whole-genome shotgun library and the fosmid library, Hallam et al. (2004) identified many genes associated with methanogenesis. Recently, a complete reverse methanogenesis pathway, including all the *mcr* subunit genes (*mcrABCDG*) and the F420-dependent 5,-10-methenyltetrahydromethanopterin reductase (*mer*) genes, has been identified in the genome of an ANME organism (Haroon et al. 2013).

However, there were repeated attempts that failed to induce reverse methanogenesis using low H_2_ and high CH_4_ concentrations (Valentine and Reeburgh 2000). Additionally, the thermodynamic yield of reverse methanogenesis is only −16 kJ mol^−1^(CH_4_), which is too low to be shared by an archaea and an SRB. Some bacterial lipids (most likely from SRB) have been found to be isotopically depleted (Thiel et al. 1999; Hinrichs et al. 2000; Pancost et al. 2000), which is difficult to explain without an interspecies carbon transfer. Moreover, instead of H_2_, many *Methanosarcinales* use acetate and methylation during methanogenesis, which were shown to be involved in AOM in some environments (Hinrichs et al. 1999). Therefore, reverse methanogenesis may not be the only process of S-DAMO. Subsequently, two new alternative mechanisms of S-DAMO were raised that have greater thermodynamic yields, involve an interspecies carbon transfer, and are identical to the results of phylogenetic analyses (Valentine and Reeburgh 2000).

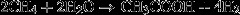
4

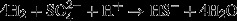
5

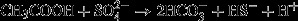
6

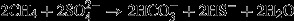
7

The first mechanism (eqs. 4–6) involves the formation of acetate and H_2_ from CH_4_ and H_2_O (eq. 4), which would then be consumed with 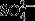 by SRB (eqs. 5 and 6). Acetate, the intermediate, transfers a carbon between CH_4_ and 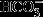. The net reaction (eq. 7) of this new mechanism is twice the reaction of reverse methanogenesis (eq. 3), which should generate twice as much free energy as the mechanism of reverse methanogenesis.

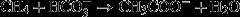
8

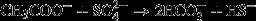
9

In the second mechanism, acetate is produced from CH_4_ and CO_2_ (eq. 8) and is consumed with 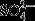 by SRB (eq. 9). Acetate is not shown in the net reaction (eq. 3) because it is an intermediate. This new hypothesis needs to be tested in future studies, but it does explain the documented findings that are inconsistent with reverse methanogenesis.

CH_4_ oxidation could not be inhibited by high pressure H_2_, which suggests that H_2_ is not an intermediate in AOM (Moran et al. 2008). Hence, Moran proposed a new model for S-DAMO that is named methylogenesis. The methylogenesis model includes two steps: the formation of methyl sulfides from CH_4_ and CO_2_ by archaea (eq. 10) and the following consumption of methyl sulfides by SRB (eq. 11). Methanethiol was concluded to play an interspecies role in AOM because the CH_4_ oxidation rates dropped 68% in the experiments treated with methanethiol (Moran et al. 2008).

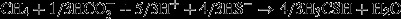
10

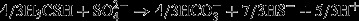
11

However, there is not a definite mechanism to account for S-DAMO in various environments, which may be due to differences in the environmental conditions and the physiological characteristics of the responsible microbes.

### The responsible microbes for S-DAMO and their peculiar properties

The type of microbes responsible for S-DAMO are termed anaerobic methanotrophs (ANME) and are represented by three different phylogenetic clusters (ANME-1, ANME-2, and ANME-3) (Hinrichs et al. 1999; Orphan et al. 2001a; Knittel et al. 2005; Schleper et al. 2005; Niemann et al. 2006). ANME-1 and ANME-2 are the most abundant groups of ANME, which are widely distributed in various anaerobic areas and produce methane, while ANME-3 archaea mostly exist in submarine mud volcanoes or occasionally in marine methane seep (Knittel and Boetius 2009; Meulepas et al. 2009). ANME-1 is divided into two subgroups, ANME-1a and ANME-1b (Knittel et al. 2005), while ANME-2 is divided into four distinct subgroups, designated ANME-2a, ANME-2b, ANME-2c, and ANME-2d (Orphan et al. 2001a,b; Mills et al. 2003). The features of these three ANME groups are summarized in Table1.

**Table 1:** Features of the three ANME groups

	ANME-1	ANME-2	ANME-3
Common features			
Habitat	Various anaerobic areas (marine sediments, cold seep, lake sediments, soils, oil field sediments, etc.)	Various anaerobic areas (marine sediments, cold seep, lake sediments, soils, oil field sediments, etc.)	Submarine mud volcanoes and marine methane seep
Subgroup	a, b	a, b, c, d	ND
Pure culture	No	No	No
Features associated with SRB			
Associated SRB	*Desulfosarcina* and *Desulfococcus*	*Desulfosarcina* and *Desulfococcus*	*Desulfobulbus*
Associated form	Often loose	Often form structured consortia	Often form structured consortia
Associated necessity	No	No	No
Single-cell form	Often	Yes	Yes
Features related to methanogens			
Related methanogen	*Methanosarcinales* and *Methanomicrobiales*	*Methanosarcinales*	*Methanococcoides*
Shape	Often rod shaped (like *Methanobacteriales* and *Methanomicrobiales*)	Often coccoid shaped (like *Methanosarcinales*)	Often coccoid shaped (like *Methanosarcinales*)
Harbour *mcrA*	Yes	Yes	Yes
* mcrA* subgroup	a, b (identified)	c, d (identified)	f (identified)
		e (possible)	
Produce methane	Yes	ND	ND
Autofluorescent under UV light (like methanogens)	Yes	Yes	Yes

ANME, anaerobic methanotrophic archaea; ND, not determined; SRB, sulfate-reducing bacteria.

These three archaeal groups of ANME are phylogenetically related to different methanogenic archaea. ANME-1 are distantly related to the orders *Methanosarcinales* and *Methanomicrobiales* (Michaelis et al. 2002; Orphan et al. 2002; Knittel et al. 2005), ANME-2 are affiliated with the order *Methanosarcinales* (Orphan et al. 2001b; Knittel et al. 2005), and ANME-3 are related to the genera *Methanococcoides* (Niemann et al. 2006; Lösekann et al. 2007; Lazar et al. 2011). The lipid structures of ANME are quite similar to those of methanogens (Elvert and Suess 1999; Hinrichs et al. 1999), and the shapes of ANME are also similar to methanogenic archaea. ANME-1 often appear as rod-shaped cells (Orphan et al. 2002), as do methanogens of *Methanobacteriales* and *Methanomicrobiales* (Lloyd et al. 2011); ANME-2 and ANME-3 often exist as coccoid-shaped cells and form clusters (Orphan et al. 2002), as do methanogens of *Methanosarcinales* (Lloyd et al. 2011). In addition, there are other remarkable similarities between ANME and methanogens. MCR, which is present in all known methanogens (Luton et al. 2002), has also been found in microbial mats that are dominated by ANME (Krüger et al. 2003); additionally, its evolutionary path in ANME mirrors that of methanogens (Hallam et al. 2003). In addition, six subgroups of *mcrA* (a, b, c, d, e, and f) among ANME archaea have been defined (Lösekann et al. 2007). Due to the presence of an F420 flavin-derived coenzyme, ANME fluoresce blue green under ultraviolet (UV) light, which is a notable characteristic of methanogens (Michaelis et al. 2002; Knittel et al. 2005; Lösekann et al. 2007). The genomes of ANME-1 and ANME-2 contain all homologous genes for enzymes associated with the canonical seven-step methanogenic pathway, although one enzyme (N^5^,N^10^-methenyl-tetrahydromethanopterin reductase) encoded by *mer* was not found in ANME-1 (Hallam et al. 2004; Meyerdierks et al. 2010). The genes, encoding the same carbon fixation pathway as methanogens, were also found in ANME-1 (Meyerdierks et al. 2010). ANME-2 have been shown to be cable to fix N_2_ (Dekas et al. 2009), as are methanogens in *Methanosarcinales* (Murray and Zinder 1984; Leigh 2000). Furthermore, ANME-1 have been shown to function as a methanogen in the methane production zone (Lloyd et al. 2011).

ANME often form consortia with SRB to catalyze S-DAMO (Fig.1A). ANME-1 and ANME-2 are associated with SRB of the *Desulfosarcina–Desulfococcus* (DSS) branch of *Deltaproteobacteria* (Boetius et al. 2000; Orphan et al. 2002), while ANME-3 are associated with SRB of the *Desulfobulbus* (DBB) branch (Niemann et al. 2006), also belonging to *Deltaproteobacteria*. ANME-1 are always loosely associated with SRB (Knittel et al. 2005), while ANME-2 and ANME-3 are usually associated with SRB forming structured consortia (Orphan et al. 2002; Niemann et al. 2006). The typical observed ANME/DSS ratio is 1:1 to 1:3 in a shell-type consortia (Boetius et al. 2000; Orcutt and Meile 2008); however, a very different ANME/DSS ratio of 7:1 was observed in hypersaline environments (Maignien et al. 2013). The ratio of ANME-3 cells to DBB cells is »1 (Lösekann et al. 2007), which differs strongly from the ANME/DSS ratio. However, a physical association with SRB is not obligatory for all three clades of ANME archaea. Most ANME-1 archaea exist as single cells or form monospecific chains without any attached partner (Orphan et al. 2002; Maignien et al. 2013). ANME-2 (Treude et al. 2005) and ANME-3 (Lösekann et al. 2007) have also been found to exist without sulfate-reducing partners. In addition, the syntrophical partners of ANME are not limited to SRB. ANME-2 are able to live syntrophically with various bacteria of *Deltaproteobacteria* as well as with *Sphingomonas* spp. of *Alphaproteobacteria* and Burkholderia of *Betaproteobacteria* (Knittel and Boetius 2009). ANME-3 have been found to occur with yet unidentified bacteria, forming mixed-type aggregates (Lösekann et al. 2007).

## Nitrate/Nitrite-Dependent Anaerobic Methane Oxidation

### Mechanism and distribution of N-DAMO

Although there were documented lines of environmental and experimental evidence of N-DAMO years ago (Smith et al. 1991; Islas-Lima et al. 2004), N-DAMO was first proposed in 2006 by Raghoebarsing et al. (2006), who discovered an n-damo enrichment culture obtained from an anoxic freshwater sediment rich in nitrate. The mechanism of N-DAMO (eq. 12) was initially hypothesized to be similar to the reverse methanogenesis of S-DAMO, which was mediated by an “ANME archaeon” (Fig.1C) with electron shuttling to denitrification (Raghoebarsing et al. 2006).

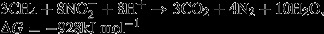
12NC10, a new bacterial candidate division, was discovered to be prevalent in the n-damo enrichment (Ettwig et al. 2008). At the same time, an archaeon affiliated with *Methanosarcinales* that was distantly related to ANME-2 (86–87%) and to methanogens (86–88%) was observed to be associated with NC10 members forming consortia. However, this archaeon was not detected in the later stages of incubation. After the apparent disappearance of the archaeon, the rate of N-DAMO did not decrease. This suggested that the archaeon was not obligatory for N-DAMO and that the process of N-DAMO was performed exclusively by members of NC10 (Ettwig et al. 2008). Then, a new “intra-aerobic” pathway of nitrite reduction was discovered based on the complete genome analysis of *Candidatus* Methylomirabilis oxyfera, the dominant bacterium affiliated with NC10, and based on isotopic labeling experiments (Ettwig et al. 2010). The new mechanism of N-DAMO suggested that 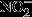 decomposes into NO and O_2_, which are mainly used to oxidize CH_4_ (Ettwig et al. 2010). The remaining O_2_ is consumed in normal respiration by terminal respiratory oxidases (Wu et al. 2011). The whole process might be exclusively mediated by *M. oxyfera*, the genome of which includes genes encoding the complete pathway for aerobic methane oxidation (Ettwig et al. 2010). The culture, including the NC10 group and the archaea partner, displayed ∼30 times higher nitrate reduction rates than the culture just including the NC10 group (Hu et al. 2009). It was suggested that the archaea might contribute significantly to the reduction of nitrate to nitrite and that the NC10 bacteria might play an important role in the reduction of nitrite (Hu et al. 2009). It is possible that *M. oxyfera* prefer to use 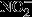 as a substrate rather than 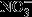. However, a high concentration of nitrite, an inhibitor to a wide range of microorganisms (Yarbrough et al. 1980), showed a toxic effect on *M. oxyfera* (Hu et al. 2011). Recently, Haroon et al. (2013) demonstrated that ANME-2d were able to independently achieve AOM (Fig.1D) via reverse methanogenesis (eq. 13) (Raghoebarsing et al. 2006) using nitrate as the terminal electron acceptor; he named the ANME-2d population *Candidatus* Methanoperedens nitroreducens and the ANME-2d lineage *Candidatus Methanoperedenaceae*.

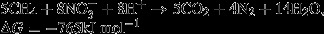
13

Even though the mechanism of N-DAMO still remains unclear, the N-DAMO process has been found to occur in different natural freshwater habitats (Raghoebarsing et al. 2006; Ettwig et al. 2008, 2009; Hu et al. 2009; Deutzmann and Schink 2011; Luesken et al. 2011a; Kampman et al. 2012; Wang et al. 2012; Yang et al. 2012; Zhu et al. 2013) where it may play an important role in the biogeochemical cycling of carbon and nitrogen.

### The microbes responsible for N-DAMO and their peculiar properties

The microbe responsible for independently coupling AOM to nitrite reduction is called *Candidatus* Methylomirabilis oxyfera; this microbe is able to reduce nitrite to dinitrogen gas without a nitrous oxide reductase (Ettwig et al. 2010). According to the ultrastructural study of *M. oxyfera* (Wu et al. 2012), there are three special aspects of *M. oxyfera*. First, the shape of *M. oxyfera* is typically polygonal (Wu et al. 2012), which is different from other bacterial shapes described (Hanson and Hanson 1996). Second, the outermost layer of the *M. oxyfera* cell consists of a putative protein surface layer (S-layer) (Wu et al. 2012) that is known to contribute significantly to mechanical cell stabilization (Engelhardt 2007). Finally, under the growth conditions used in the ultrastructural study, *M. oxyfera* did not develop intracytoplasmic membranes (ICMs), which are an ultrastructural feature shared by most methanotrophs (Wu et al. 2012). In addition, the *M. oxyfera* genome contains a complete *pmo* gene cluster for aerobic methane oxidation (Ettwig et al. 2010), but the genetic analyses of different *M. oxyfera* enrichment cultures showed that they formed a distinct group affiliated with the *pmoA* genes of aerobic methanotrophs (Luesken et al. 2011b).

In the N-DAMO enrichment culture, the archaea partner of the NC10 bacteria was subsequently named *Candidatus* Methanoperedens nitroreducens (Haroon et al. 2013). *Candidatus* Methanoperedens nitroreducens is able to use nitrate instead of nitrite as the terminal electron acceptor, which is different from *M. oxyfera* (see Table2) (Ettwig et al. 2010). The N-DAMO pathway of *M. nitroreducens* is proposed as the reverse of methanogenesis because the genome of *M. nitroreducens* includes all *mcr* subunit genes (*mcrABCDG*) and F420-dependent *mer* genes for a full reverse methanogenesis (Haroon et al. 2013). In addition, owing to the existence of a full reductive acetyl-CoA (carbon fixation) pathway and acetyl-CoA synthetase in *M. nitroreducens* (Haroon et al. 2013), it was predicted that *M. nitroreducens* might be capable of producing acetate, as was suggested for ANME-1 (Meyerdierks et al. 2010). *Candidatus* Methanoperedens nitroreducens is a new species responsible for N-DAMO, so more studies are needed regarding this new member of N-DAMO in the future.

**Table 2:** Comparisons between *Candidatus* Methylomirabilis oxyfera (*M. oxyfera*) and *Candidatus* Methanoperedens nitroreducens (*M. nitroreducens*), the two known species responsible for N-DAMO

Features	*M. oxyfera*	*M. nitroreducens*
Habitat	Freshwater environments	Freshwater environments
Pure culture	No	No
Kingdom	Bacteria	Archaea
Affiliated microbes	NC10	ANME-2d
Shape	Atypical polygonal	Irregular coccus
Growth conditions	Anaerobic	Anaerobic
Produce O_2_	Yes	No
Electron acceptor (N-DAMO)	Nitrite	Nitrate
Mechanism hypothesis (N-DAMO)	Aerobic methane oxidation	Reverse methanogenesis
Related gene (N-DAMO)	*pmo* gene cluster	*mcr* gene cluster

ANME, anaerobic methanotrophic archaea; N-DAMO, nitrate/nitrite-dependent anaerobic methane oxidation.

## Metal Ion (Mn^4+^ and Fe^3+^)-Dependent Anaerobic Methane Oxidation

Similar to the sulfate-dependent mode, manganese (Mn^4+^) (eq. 14) and iron (Fe^3+^) (eq. 15) can be used as electron acceptors of AOM in marine methane-seep sediments (Beal et al. 2009). This new pathway that involves coupling AOM with metal ion reduction is called M-DAMO.

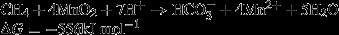
14

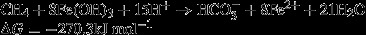
15

An uncultivated group, affiliated with the marine benthic group D (MBGD) (Fig.1B), was found to be the most abundant microorganisms in the sediment of M-DAMO (Beal et al. 2009). ANME-1 and ANME-2 were also identified, while a small percentage of ANME-3 were observed only in the subsequent manganese incubations (Beal et al. 2009). Although the mechanism of M-DAMO and the responsible microbes involved still remains unclear, M-DAMO may play an important role in global marine AOM because of the large amounts of manganese and iron provided to continental margins from rivers (Beal et al. 2009).

## Discussion of Key Issues

In the light of recent progress regarding AOM, several unclear issues need to be further elucidated. These issues are discussed below.

### Is ANME-1 a hypersaline anaerobic methanotroph ecotype?

The ANME population consisting only of ANME-1 was first found in a natural sedimentary that was high in salt (Lloyd et al. 2006). Then, ANME-1 was reported in other hypersaline environments (Yakimov et al. 2007; Cono et al. 2011; Maignien et al. 2013). Therefore, ANME-1 may be a hypersaline anaerobic methanotroph ecotype, which was suggested to be related to the comparatively low effect of ionic strength on ANME-1 (Maignien et al. 2013). ANME-1 cell membranes contain high contents of glycerol dialkyl glycerol tetraethers (GDGTs) (Niemann and Elvert 2008), which are characterized by a lower permeability compared with typical membrane lipids (Yamauchi et al. 1993; Valentine 2007). ANME-2 and ANME-3 cell membranes contain less or no GDGTs; the dominant component of ANME-2 and ANME-3 cell membranes is diethers exhibiting a higher permeability (Rossel et al. 2011). In addition, the ANME-1 genome was shown to contain genes coding for mannosylglycerate and di-myo-inositol-phosphate synthesis pathways (Meyerdierks et al. 2010), which are widely used to increase the turgor pressure by halophilic microorganisms (Roberts 2004; Empadinhas and da Costa 2008). Recently, proteins involved in gas vesicle synthesis have been identified in the proteome of ANME-1 (Stokke et al. 2012). Gas vesicles have also been observed in halophilic archaea (Walsby 1994), which might function in a salt stress response (Hechler and Pfeifer 2009). The above may contribute to the domination of ANME-1 in hypersaline environments. More needs to be investigated on this topic in the future.

### The effect of oxygen on *M. oxyfera*

*Candidatus* Methylomirabilis oxyfera has the ability to conduct methane oxidation through a strictly O_2_-dependent reaction catalyzed by particulate methane monooxygenase (pMMO) (Ettwig et al. 2010). However, it was found that the addition of either 2% or 8% of O_2_ had an overall detrimental effect on *M. oxyfera*, and the ability of this bacterial species did not resume the original level (Luesken et al. 2012). These observations suggest that the O_2_ production and consumption of *M. oxyfera* is tightly controlled process, and the detrimental effect of O_2_ on *M. oxyfera* may be unrecoverable. However, most *M. oxyfera* and *M. oxyfera*-like bacteria have been observed in the oxic/anoxic interface of freshwater habitats (Raghoebarsing et al. 2006; Ettwig et al. 2008; Hu et al. 2011; Luesken et al. 2011a,b). Additionally, it is possible that the applied oxygen concentration was too high. In consideration of the above information, it is controversial whether *M. oxyfera* could use external O_2_ to oxidize methane. The effect of oxygen on *M. oxyfera* still remains unclear.

### The relationship of *M. nitroreducens* with ANME

*Candidatus* Methanoperedens nitroreducens, a new species responsible for N-DAMO (Haroon et al. 2013), which is affiliated with ANME-2d, is the fourth subgroup of ANME-2 for S-DAMO (Mills et al. 2003). In addition, a full reductive acetyl-CoA (carbon fixation) pathway and acetyl-CoA synthetase have been identified in *M. nitroreducens*. It was predicted that *M. nitroreducens* might be able to produce acetate (Haroon et al. 2013), as can ANME-1 (Meyerdierks et al. 2010). The reported relationship of *M. nitroreducens* with ANME suggests that N-DAMO may be associated with S-DAMO, which warrants further investigation.

## Conclusion

The microbes responsible for AOM are difficult to cultivate because of their low growth rates (Jagersma et al. 2009). AOM is an “active” process in microbial studies that contributes significantly to the global methane cycle. Currently, three different processes (Table3) are thought to be responsible for AOM, with sulfate, nitrite/nitrate, and metal ions (Mn^4+^ and Fe^3+^) serving as electron acceptors. However, the specific mechanism of AOM is not fully known, and the exact features of the responsible microbes require further study.

**Table 3:** Comparisons between the three processes of AOM: S-DAMO, N-DAMO, and M-DAMO

Features	S-DAMO	N-DAMO	M-DAMO
Habitat	Marine environments and freshwater environments	Freshwater environments	Marine environments
Mechanism hypothesis	Reverse methanogenesis, acetogenesis, and methylogenesis	Aerobic methane oxidation and reverse methanogenesis	ND
Electron acceptor	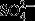	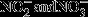	Mn^4+^ and Fe^3+^
Responsible microbes	ANME	*M. oxyfera* and *M. nitroreducens*	MBGD (possible)
Reaction (AOM)	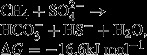 (eq. 3)	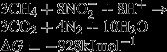 (eq. 12) and	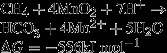 (eq. 14) and
		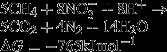 (eq. 13)	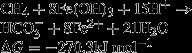 (eq. 15)

S-DAMO, sulfate-dependent anaerobic methane oxidation; N-DAMO, nitrate/nitrite-dependent anaerobic methane oxidation; M-DAMO, metal ion (Mn^4+^ and Fe^3+^)-dependent anaerobic methane oxidation; ANME, anaerobic methanotrophic archaea; *M. oxyfera*, *Candidatus* Methylomirabilis oxyfera; *M. nitroreducens*, *Candidatus* Methanoperedens nitroreducens; MBGD, marine benthic group D; ND, not determined; AOM, anaerobic oxidation of methane.
